# Specific Cutting Forces of Isotropic and Orthotropic Engineered Wood Products by Round Shape Machining

**DOI:** 10.3390/ma11122575

**Published:** 2018-12-18

**Authors:** Giacomo Goli, Rémi Curti, Bertrand Marcon, Antonio Scippa, Gianni Campatelli, Rocco Furferi, Louis Denaud

**Affiliations:** 1 GESAAF—University of Florence, Via S. Bonaventura 13, IT-50145 Florence, Italy; giacomo.goli@unifi.it; 2 LaBoMaP—Arts et Métiers Paristech, Rue Porte de Paris, FR-71250 Cluny, France; bertrand.marcon@ensam.eu (B.M.); louis.denaud@ensam.eu (L.D.); 3 DIEF—University of Florence, Via Santa Marta 3, IT-50139 Florence, Italy; antonio.scippa@unifi.it (A.S.); gianni.campatelli@unifi.it (G.C.); rocco.furferi@unifi.it (R.F.)

**Keywords:** cutting forces, wood, wood based materials, machining, routing, specific cutting coefficient, LVL, MDF, poplar, beech

## Abstract

The set-up of machining parameters for non-ferric materials such as wood and wood-based materials is not yet defined on a scientific basis. In this paper, a new rapid experimental method to assess the specific cutting coefficients when routing isotropic and orthotropic wood-based materials is presented. The method consists of routing, with different depths of cut, a given material previously machined to a round shape after having it fixed on a dynamometric platform able to measure the cutting forces. The execution of subsequent cuts using different depths of cut allows the calculation of the specific cutting coefficients. With the measurement being done during real routing operations, a method to remove machine vibrations was also developed. The specific cutting coefficients were computed for the whole set of grain orientations for orthotropic materials and as an average for isotropic ones. The aim of this paper is to present and validate the whole method by machining selected materials such as Polytetrafluoroethylene—PTFE (isotropic), Medium Density Fiberboard—MDF (isotropic), beech Laminate Veneer Lumber—LVL (orthotropic) and poplar LVL (orthotropic). The method and the proposed analysis have been shown to work very effectively and could be used for optimization and comparison between materials and processes.

## 1. Introduction

The determination of the cutting forces when machining wood and for Engineered Wood Products (EWPs) is a topic that has been discussed since 1950. A well-known pioneering work was conducted in 1950 by Kivimaa [1] who developed a testing apparatus and investigated the effect of different variables on the cutting forces, giving important conclusions on the effect of cutting speed, chip thickness, grain orientation, wood density and tool geometry. Many authors deepened these aspects in the following years and an excellent summary of these research activities was reported in the reviews written by Marchal et al. [2], as an output of the COST Action E35, and by Naylor and Hackney [3]. A lot of work was done to study the cutting forces and cutting power in the principal working directions described, respectively, as A, B and C by Kivimaa [1] and 90-90, 90-0 and 0-90 by McKenzie [4]. Much research was then conducted in simplified cutting conditions such as the experiments performed on dynamic pendulum by Eyma [5] or by orthogonal cutting at low and high speed by Wyeth et al. [6]. Furthermore, real cutting conditions were tested using cinematically simple methods such as band-sawing, for example, from Orlowski et al. [7] and Moradpour et al. [8].

Additionally, the influence of grain orientation on the machining of wood in terms of cutting forces and final quality has already been studied by different authors. Fundamental work was published by Stewart [9] on the effect of the grain orientation on several properties such as cutting forces and final quality. Other work about the effect of machining at different grain orientations on the cutting forces and on the acoustic emissions count was done by Cyra et al. [10]; on the chip formation and cutting forces when routing by Goli et al. [11,12]; on the chip formation and chip type when orthogonally cutting at high speed by Wyeth et al. [6]; and when orthogonally cutting green beech wood by Curti et al. [13]. In these works, the authors highlight the effect on cutting forces when machining with different grain orientation. However, they used time-consuming experimental procedures and in the most cases used simplified processes. The sole investigation on cutting forces evolution for a whole 360° grain orientation range over one single experiment was conducted by Costes et al. [14]. However, this study was much closer to a turning operation than to a regular milling configuration, with the limit of a very low cutting speed.

The execution of these measurements during real machining operations involving rotating tools is also a subject that requires further investigations. Rotating tools produce a cyclic impact of the cutting edge on the piece to be machined that results in dynamic excitations leading the system piece-support-tool-machine to vibrate. The result is a strong alteration of the force signal that is frequently much lower than the noise. In order to provide an interpretation of these data, two main strategies can be adopted: low pass filtering and dynamic compensation. Low pass filtering can considerably reduce the noise but also affects the force signals—it can then be considered as a simple method to compare signals as in Goli et al. [11]; however, it was not as suitable a method to determine specific cutting coefficients to be used for the calculation of the cutting forces. Dynamic compensation requires the assessment of the transfer function able to transform the output of the system into the original input. This method, if applicable, allows to precisely determine the cutting forces as shown for metal cutting by Scippa et al. [15]; however, as far as we know, it has not yet been applied to the wood machining sector. In this work, the samples were machined to a round shape according to the method proposed by Goli and Sandak in [16] and the forces were measured via a dynamometer. The method has already given interesting results for the comparison of final surface quality, for example, by Goli et al. [17]. The cutting forces were acquired and both filtering and dynamic compensation approaches were attempted. Filtering gave good results but without a physical sense of the resulting coefficients because of the filtering procedure. Dynamic compensation was also attempted but without success because of the high level of noise and because of the measurement of too few samples during the cut of a single chip. Finally, a method based on a moving average was successfully applied to calculate the cutting forces.

## 2. Materials and Methods

Different EWPs were machined to a round shape with the method proposed by Goli and Sandak [16]. In addition, prior to machining, the disk was rigidly fixed by four screws on a Kistler 9255A (KISTLER Group, Winterthur, Switzerland) type tri-axial piezoelectric dynamometer to measure the cutting forces. The Kistler 9255A has a 260 × 260 mm^2^ top plate and a measurement range going from −20 to +20 kN on the X- and Y-axes used to acquire the cutting forces in the experiment. The threshold of the instrument is 0.01 N. Its natural frequency is around 1.5 kHz for both X- and Y-axes. The dynamometer was driven by a Kistler type 5019 charge amplifier set with a 100 N·V^−1^ gain and “Long” time-constant mode (i.e., drift dominates any time-constant effect). The signals were acquired after low pass filtering at 10 kHz in order to avoid aliasing. The disks were kept lifted above the platform plate by a 10 mm-thick piece of phenolic beech plywood with a smaller radius than the disk so as to have only a peripheral contact between the tool and the target material during machining.

The disk was machined using a 3-axis Numerical Control (NC) machine Record 1 (SCM Group, Rimini, Italy). The initially squared sample was made round by a spiral router bit with a chip-breaker. Once the sample was machined to a round shape, the surface was prepared by preliminary cuts in order to ensure the same initial conditions before doing the finishing cut and acquiring the cutting forces. Between the preparation cuts and the finishing cut, the disk was not disassembled so as not to introduce positioning errors. Both of these operations were done by a single straight blade balanced tool in order to obtain a constant chip thickness not influenced by multi blade tool run-off or blade misalignment. The diameter of the tool was 80 mm, and the blade was a tungsten carbide freshly sharpened insert with a rake angle of 25° and a blade angle of 55°. The surface preparation was done by five successive cuts at a spindle speed of 3000 rpm, a feeding speed of 1 m min^−1^, and a radial depth of cut (reduced to “depth of cut” in the following) of 0.2 mm. The low feed speed and the low depth of cut allowed us to obtain a very thin chip thickness and to reduce as much as possible the formation of defects on the surface; those settings allowed us to avoid the influence of the preparation process on the measurements to be taken during the finishing.

**Figure 1:**
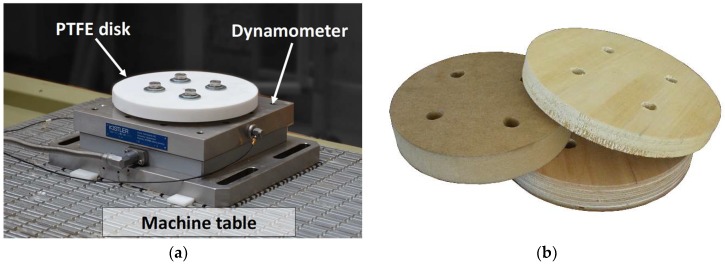
(**a**) Polytetrafluoroethylene (PTFE) disk after the preparation cuts installed on the dynamometer. The dynamometer is held on the machine table by a conventional vacuum suction system; (**b**) Medium Density Fiberboard (MDF), Beech LVL and Poplar Laminate Veneer Lumber (LVL) disks used in the tests.

For the finishing (when the cutting forces were measured), a spindle rotation of 3000 rpm and a feeding speed of 2 m min^−1^ were set. The measurements were taken by machining different wooden species and EWPs with up- and down-milling techniques, with 0.3, 0.7, 1.1 and 1.5 mm of depth of cut. These depth of cut values, in the given machining setup when finishing, resulted in average chip thicknesses ($h_{m}$) of 0.041, 0.062, 0.078 and 0.091 mm. During the finishing cut, the analogic outputs of the charge amplifiers were acquired synchronously with a sampling frequency of 50 kHz by a 16-bit National Instrument acquisition board model NI−9215 (National Instruments, Austin, TX, USA) installed on a USB chassis model cDAQ-9174. The different channels were acquired by DASYLab^®^ (Measurement Computing Corporation, Norton, MA, USA) software and analyzed with MATLAB^®^ (MathWorks, Natick, MA, USA). Polytetrafluoroethylene (PTFE), being isotropic and homogeneous, was machined as the reference material while the Medium Density Fiberboard (MDF) was machined as the most equivalent to isotropic material within the EWPs. Beech Laminate Veneer Lumber (LVL) and Poplar LVL were machined as orthotropic EWPs with two very different densities. The different materials were machined after a stabilizing period in an internal environment resulting in a homogeneous moisture content measured to be about 9%. The moisture content during machining and the main physical properties of the materials used at 20 °C and 65% of moisture content are reported in Table 1. The PTFE disk installed on the dynamometer above the machine table is shown in Figure 1a while the MDF, beech LVL and Poplar LVL disks are shown in Figure 1b.

**Figure 2:**
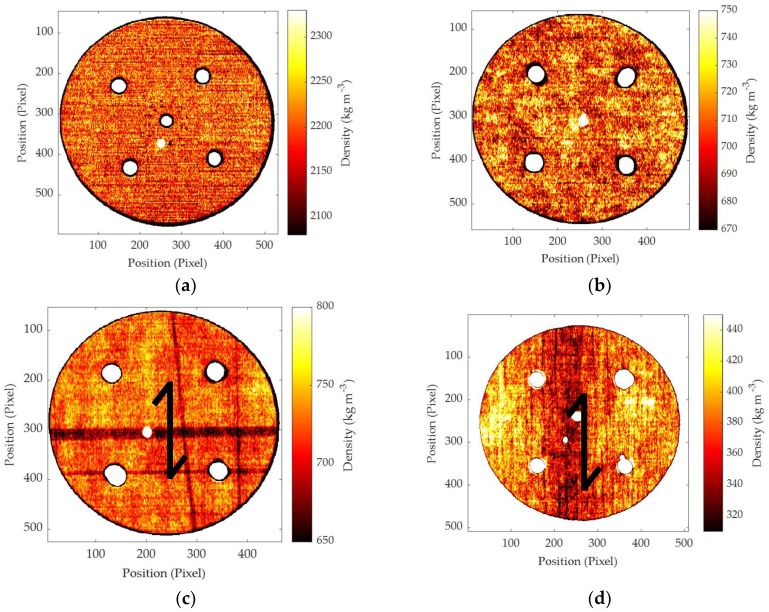
Densities of the machined samples measured by X-ray densitometry: (**a**) PTFE sample; (**b**) MDF sample; (**c**) beech-LVL sample; (**d**) poplar-LVL sample. Grain direction is reported for beech and poplar LVL.

In order to check the system and compare the results of the data processing with well-known expectations, a first test on a PTFE disk was done. PTFE is, in fact, very isotropic, homogeneous and an easy to cut material. In order to be sure of the homogeneity of the PTFE disk, and prevent any influence of density variation in the measurements, an X-ray scan was done on the rough disk before machining. As shown in Figure 2a, the density of PTFE can be considered quite constant with a variation range of ±2.5% around the mean value. Mean values are reported in Table 1. The same test was applied to the other materials to be machined, showing a good homogeneity for MDF (Figure 2b) with a variation range of ±3.5%; few zones with a missing section of veneer can be observed for beech LVL (Figure 2c) with a variation range of ±7.5% and a quite homogeneous general density; finally, a larger variation of ±17% can be observed for poplar LVL, showing a gradual increase from the exterior to the central part, moving orthogonally to the grain. While for PTFE, MDF and beech LVL, the density variation can be considered as not influencing the cutting forces; in the case of poplar LVL, higher forces than expected could be measured when machining perpendicular to the grain.

**Figure 3:**
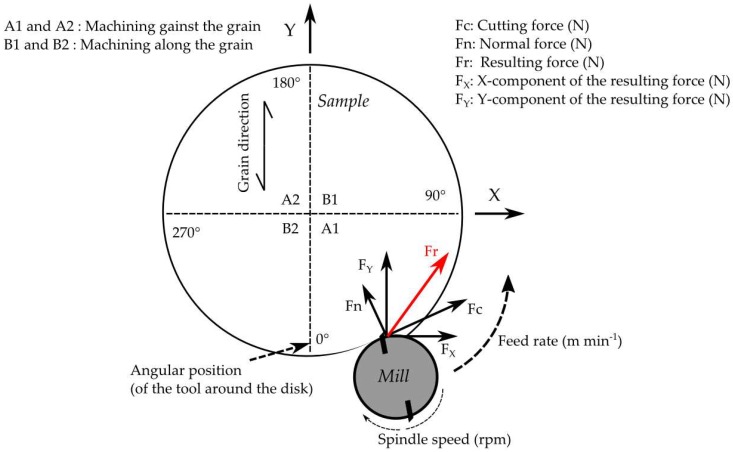
Bi-dimensional scheme of peripheral milling of a disk with up-milling technology. Grain orientation is only relevant for orthotropic materials such as solid wood or LVL: 0° grain orientation corresponds to machining across the grain; from 1 to 89° corresponds to machining against the grain; 90° corresponds to machining along the grain; 91 to 179° corresponds to machining with the grain, and 180° corresponds to machining across the grain again (as 0°). The machining of the sector A2 is a replica of the machining of the sector A1, and in the same way, the sector B2 is a replica of the machining of the sector B1.

The rough disk was then fixed on the dynamometric platform and the preparation cuts were executed. Finally, the disk was machined at the desired depth of cut with the machining process schematized in Figure 3, and the cutting forces acquired.

**Figure 4:**
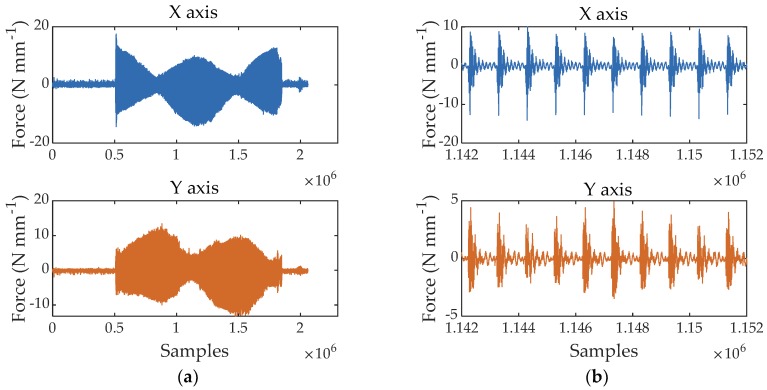
Rough cutting forces measured by the dynamometer X- and Y-axes when up-milling a PTFE disk with a depth of cut of 1.5 mm: (**a**) full signals; (**b**) same signals magnified over 10 tool revolutions. The forces are normalized by the sample thickness.

Figure 4a shows the rough forces for the X- and Y-axes acquired when machining the PTFE disk. The forces are already normalized by the disk thickness, and thus, are expressed in N mm^−1^ of blade length engaged in the cutting process. Figure 4b shows a magnification of the rough signal corresponding to 10 cutting periods where a high dynamic excitation can be observed as a dumped free vibration superposed to the force signal. The milling with one blade is a highly discontinuous process explaining why the whole system (wood disk + dynamometer + machine) is strongly solicited dynamically to vibrate at its natural frequency that was experimentally found to be about 1.5 kHz.

**Figure 5:**
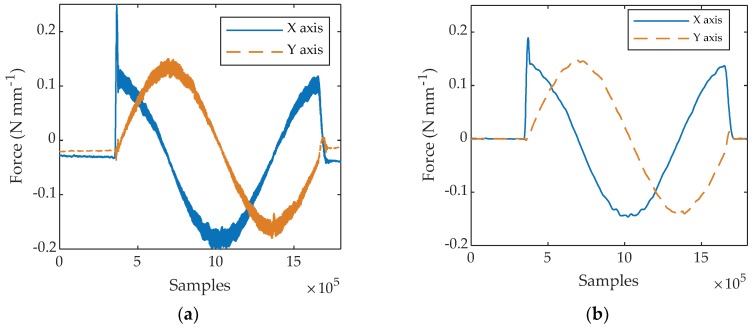
PTFE-machined up-milling with a depth of cut of 1.5 mm forces signals: (**a**) after applying a moving average on 10 cutting periods; (**b**) after applying a moving average on 10 cutting periods and a smoothing on 20 cutting periods. The signals after smoothing were also detrended.

In order to remove the undesired vibrations, different post-processing approaches were attempted, such as low pass filtering, dynamic compensation and a simple method based on the moving average. Low pass filtering is suitable to remove high frequency vibrations, however, unfortunately the filtering process has several spurious effects on the signal, and the different cut-off frequencies may produce different outputs. For these reasons, low pass filtering was considered not suitable to have a proper estimate of the cutting forces. Dynamic compensation, as applied by Scippa et al. [15], is a very effective method to correct the influence of the system’s dynamics on the measured signals. Unfortunately, in this case, the dynamic behavior of the whole system was complex and difficult to mathematically model in order to apply this method. Therefore, to remove the undesired vibrations, the rough signals were processed with a moving average calculated on a window of 10 cutting periods. With the integral of the periodic signal being equal to zero, it is possible to remove the undesired vibrations of the machine tool-dynamometer system, keeping only the mean cutting forces averaged on the cutting period. In Figure 5a, the effect of the moving average can be observed. As can be seen, most of the noise is easily removed and clear trends can be observed. A high reduction of the normalized forces can also be observed passing from a maximum value of about 12 N mm^−1^ for the un-averaged signal to a value of 0.12 N mm^−1^ for the averaged signal, with a reduction ratio of 100. This highlights how most of the signal depends on resonances rather than on the forces themselves. After having applied the moving average, in order to completely remove the noise, the signal was smoothed with a span of 20 cutting periods and drifting linear trends were removed. These data operations are visible by comparing Figure 5a,b.

The initial and final part of the signals were then removed in order to keep only the cutting forces during the disk machining and the resultant force was computed according to Equation (1):

(1) $$Fr=\sqrt{F_{X}^{2}+F_{Y}^{2}}.$$

**Figure 6:**
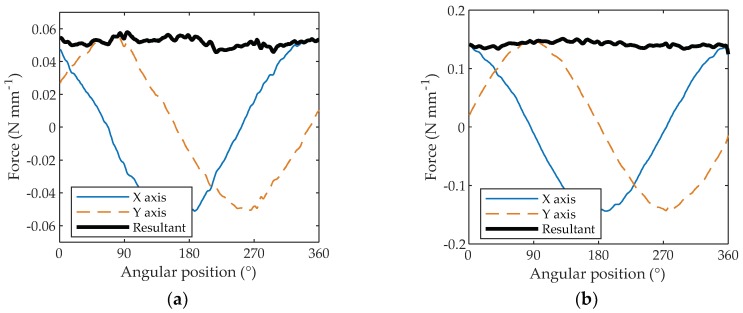
Resultant force on PTFE-machined up-milling with a depth of cut of: (**a**) 0.3 mm; (**b**) 1.5 mm.

The data were also thinned by averaging the samples for every degree of angular position passing from thousands of samples to 360 samples on 360°. Figure 6a shows the forces for every degree of angular position on the X- and Y-axes and the resultant force acquired during the machining of a disk, after applying the moving average, the smoothing and the detrending. The initial and final zones with the machine turning idle were removed. Figure 6a shows the average X, Y and R forces when up-milling PTFE with 0.3 mm of depth of cut and Figure 6b shows the forces up-milling PTFE with 1.5 mm of depth of cut. As can be observed, the resultant force is a quite steady horizontal line, showing how the resulting force obtained by machining an isotropic material is not influenced by the angular position as expected. It is also clear how machining with different depths of cut (0.3 and 1.5 mm) produces different resultant forces (~0.05 N mm^−1^ for 0.3 mm of depth of cut and ~0.13 N mm^−1^ for 1.5 mm of depth of cut).

In order to convert *Fr* into cutting force (Fc) and normal force (Fn), it is essential to know the direction of the resultant force. The resultant force direction ($Fr_{angle}$), was computed according to Equation (2):

(2) $$Fr_{angle}=\arctan\left(F_{X},F_{Y}\right).$$

**Figure 7:**
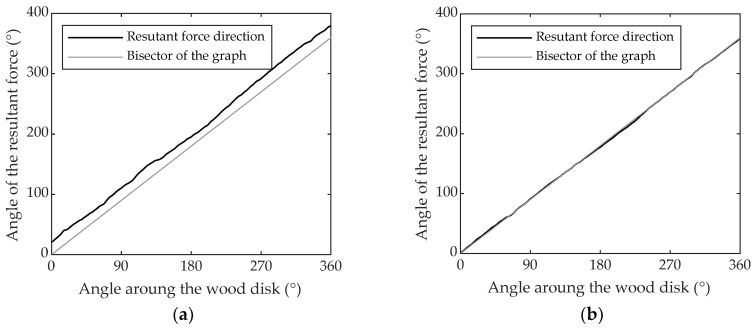
Resultant force angle on PTFE-machined up-milling with a depth of cut of: (**a**) 0.3 mm; (**b**) 1.5 mm.

The resultant force direction computed when up-milling a PTFE disk with a depth of cut of 0.3 mm, is shown in Figure 7a. As can be observed, the $Fr_{angle}$ is linear and parallel to the graph bisector, showing how, for an isotropic material, the resultant force of the forming chip always has the same direction towards the piece surface being machined. In Figure 7b, $Fr_{angle}$ is shown when up-milling a PTFE disk with a depth of cut of 1.5 mm. This also shows how, for a different depth of cut, the absolute value is different, because for the same angle the resultant force has a different direction; however, the resultant force turns 360°around the piece of wood once again.

Once Fr and $Fr_{angle}$ are computed, the force can be decomposed into the cutting force (Fc) and the normal force (Fn) according to Equations (3) and (4):

(3) $$Fc=Fr\cdot \cos\left(Fr_{angle}\right),$$

(4) $$Fn=Fr\cdot \sin\left(Fr_{angle}\right).$$

**Figure 8:**
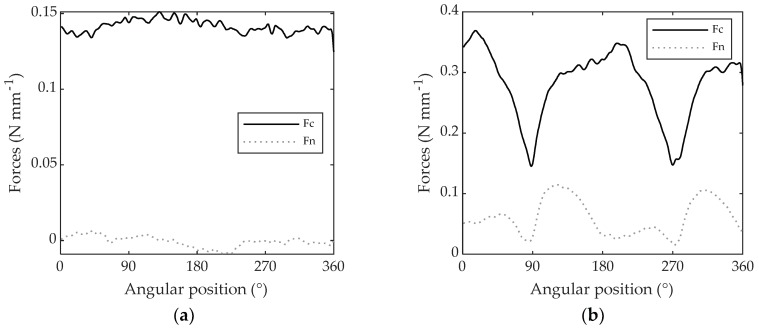
Cutting force and normal forces in up-milling with a depth of cut of 1.5 mm machining: (**a**) PTFE; (**b**) beech LVL.

The cutting and normal forces, when up-milling PTFE (isotropic) and Beech LVL (orthotropic) disks with a depth of cut of 1.5 mm, are shown in Figure 8a,b, respectively.

It can be observed how Fc and Fn are constant on the whole PTFE disk while they vary depending on the grain orientation between ~0.15 N mm^−1^ and 0.37 N mm^−1^ for Fc and ~0.02 N mm^−1^ and 0.12 N mm^−1^ for Fn when machining beech LVL. Forces are at minimum when machining parallel to the grain (90° and 270°) and maximum when machining perpendicular to the grain (0°, 180° and 360°). The calculation of the specific cutting coefficients, as reported in [18], is done on the Fc component, because Fn will not be analyzed in this study.

As can be observed in Figure 8b, when machining a disk, the tool to grain interaction is repeated twice. On one disk, there are two replicas of the same experiment, going from 0° to 180° and going from 180° to 360°. The results from the two half-disks were very similar. Using the moving average as a method to remove noise worked very effectively but the result of this procedure was that an average force, referring to 20 periods, was obtained. Because the cutting conditions did not vary significantly in over 20 tool cuts, we can suppose that the average of 20 cuts was the same as one cut. This average value can refer to a cutting period that in our case, working with only one blade, is a complete tool revolution. This average force per revolution (Fc) can be transformed in the average force ($\bar{Fc}$) that refers to the period during which the tool was engaged inside the material. This can be done by a geometric computation with the whole circumference referring to the work angle ($\varphi_{s}$) as in Equation (5):

(5) Fc¯=Fcφs 2π.

**Figure 9:**
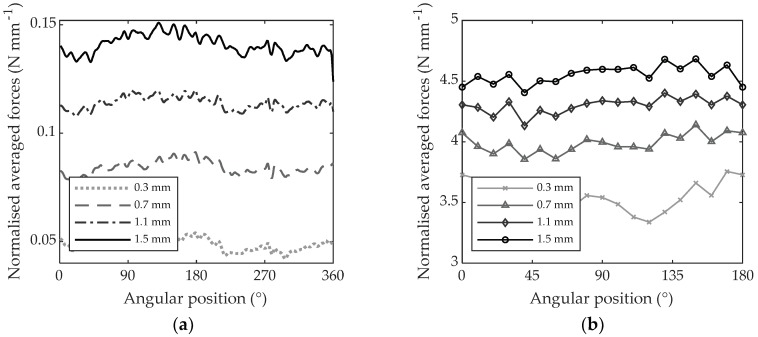
Cutting forces machining with different depths of cut (0.3, 0.7, 1.1 and 1.5 mm): (**a**) average force per revolution for every degree of angular position (Fc); (**b**) average cutting force referring to the cutting period reported every 10° of angular position ($\bar{F_{C}}$).

Figure 9a reports Fc when up-milling PTFE with depths of cut of 0.3, 0.7, 1.1 and 1.5 mm for the whole angular range of 360°. Figure 9b reports $\bar{Fc}$ as computed applying Equation (5). Because the cutting from 180° to 360° is a repetition of the cutting from 0° to 180°, the vector of the forces was divided into two halves and the two halves were averaged for the different grain orientations. To improve clarity, starting from Figure 9b, only the values of forces every 10° of angular position will be visualized.

**Figure 10:**
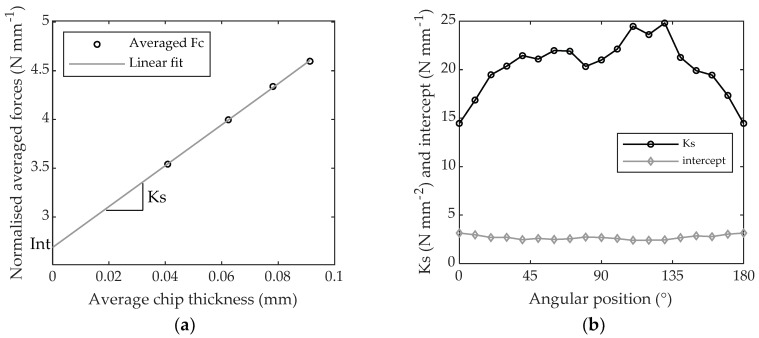
Specific cutting coefficient (Ks) and intercept when machining PTFE: (**a**) with 0.3, 0.7, 1.1 and 1.5 mm of depth of cut computed at 90° grain orientation; (**b**) gathered for every 10°-grain orientation.

Once the values of ($\bar{Fc}$) were extracted every ten degrees, they were plotted against chip thickness and a linear regression was applied in order to calculate the slope and the intercept (*Int*) of the linear regression (Figure 10a). The slope corresponds to the specific cutting coefficient Ks. Values of Ks and intercepts are displayed in Figure 10b for the whole 180° grain orientation range. Their average value, standard deviation, minimum and maximum values on this range are gathered in Table 2.

The same mathematical analysis presented for PTFE was applied on EWPs: MDF, beech LVL and poplar LVL. MDF was chosen because it is very homogeneous and isotropic and enables a comparison of the results with those of PTFE. Beech and poplar LVL were chosen as the orthotropic materials. They were chosen as they are known to be quite homogeneous materials (compared to classical conifers for instance); this minimizes the effect of annual rings. LVL was chosen instead of solid wood because it represents an almost perfectly tangential sample.

## 3. Results

The same mathematical approach, once validated for PTFE, was applied to the selected EWPs. The results are presented in the following section.

### 3.1. MDF

**Figure 11:**
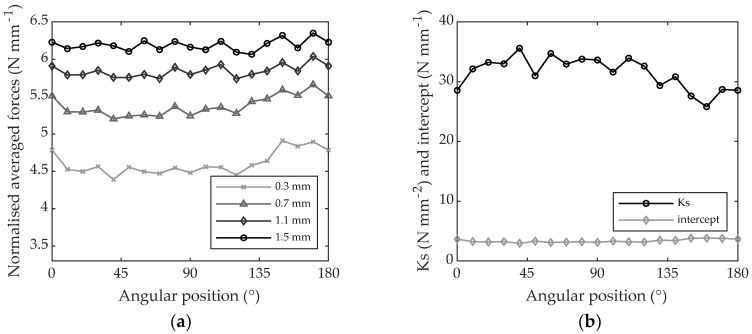
(**a**) Average cutting force referring to the cutting period versus angular position when machining MDF with 0.3, 0.7, 1.1 and 1.5 mm of depth of cut; (**b**) intercept and specific cutting coefficient gathered every 10° of angular position.

The results obtained for MDF are very close to those obtained when machining PTFE in terms of constant forces plotted against angular position, as shown in Figure 11a. The specific cutting coefficient, presented in Figure 11b, is very stable as well within the grain orientation, with little variation (2.68 N mm^−2^ of standard deviation for a mean value of 31.44 N mm^−2^) as shown in Table 3.

### 3.2. Beech LVL

**Figure 12:**
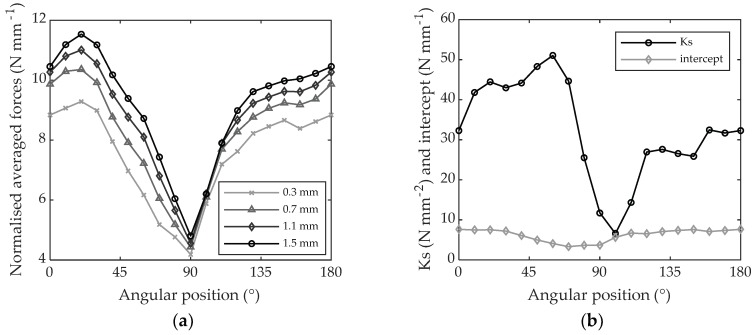
(**a**) Average cutting forces referring to the cutting period versus angular position when machining beech LVL with 0.3, 0.7, 1.1 and 1.5 mm of depth of cut; (**b**) intercept and specific cutting coefficient gathered every 10° of angular position.

Beech LVL is an orthotropic material and therefore the cutting forces (when machining the wood disk) are largely influenced by the angular position, i.e., by the grain orientation, as shown in Figure 12a. As can be observed, the normalized cutting forces are almost two times larger when machining across the grain (i.e., 0 and 180° of angular position) than when machining along the grain (i.e., 90° of angular position). The effect of the radial depth of cut, as shown in Figure 12a, is clear and higher depths of cut correspond to higher cutting forces. The cutting forces, in general, are also higher when machining against the grain (i.e., 1–89°) than with the grain (i.e., 91–179°), and a higher increase in forces with the increase in depth of cut can be observed in the first zone; this corresponds to a higher $K_{s}$ in the first zone, as shown in Figure 12b. The minimum $K_{s}$ can be found at a value of 100° of grain orientation and the maximum at 60° of grain orientation with very large variations from 6.61 N mm^−2^ to 51.04 N mm^−2^. The intercept variation is much lower, displaying values from 3.33 N mm^−1^ to 7.66 N mm^−1^. Ks and intercept values for every 10° of grain orientation are reported in Table 4.

### 3.3. Poplar LVL

**Figure 13:**
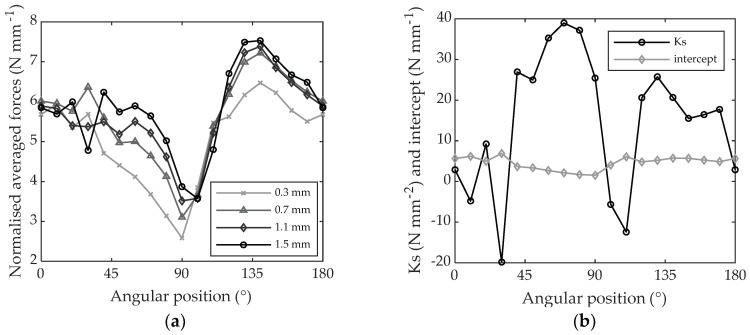
(**a**) Average cutting force referring to the cutting period versus angular position when machining poplar LVL with 0.3, 0.7, 1.1 and 1.5 mm of depth of cut; (**b**) intercept and specific cutting coefficient gathered every 10° of angular position.

Similar to beech LVL, poplar LVL is an orthotropic material and the cutting forces when machining the disks are largely influenced by the grain orientation as shown in Figure 13a. As can be observed, the normalized cutting forces are once again about 2 times larger when machining across the grain (i.e., 0 and 180° of angular position) than when machining along the grain (i.e., 90° of angular position). The effect of increasing the radial depth of cut, as shown in Figure 13a, is that the force needed to cut the chip is generally increased; however, for poplar LVL this cannot be generalized. There are some situations where the increase in depth of cut does not seem to have any effects on the cutting forces, for example, for the angular positions going from 0 and 20° and from 100° to 110°. Additionally, the consequences on the cutting forces were moderate for angular positions going from 120° to 170°. This corresponds to a higher Ks in the angular zone going from 40° to 90° and from 120° to 170° as shown in Figure 13b. The minimum Ks was identified at 110° of angular position because the valley at 30° was considered a measurement error resulting only from the very low force value measured for 1.5 mm of depth of cut (see Figure 13a). The maximum was found at 70° of grain orientation with very large variations from −12.41 N mm^−2^ to 38.98 N mm^−2^. The intercept variation is much lower, from 1.54 N mm^−1^ to 6.90 N mm^−1^.$\;K_{S}$ and intercept values for every 10° of grain orientation are reported in Table 4.

## 4. Discussion

The test performed on PTFE shows that the proposed method of machining, force measurement, assessment and data processing works well to assess the cutting forces and, above all, to evaluate the specific cutting coefficient for different grain orientations in a single machining operation. This is also confirmed by the results when machining isotropic and orthotropic materials that are in line with the author’s expectations and literature sources. Isotropic materials show constant values of cutting forces for all angular positions while orthotropic materials show very different levels of cutting forces depending on the angular position and consequently on the grain orientation. The behavior of forces, increasing the depth of cut, and consequently, the chip thickness, is in line with the expectation with higher cutting forces for higher depths of cut. For denser products, such as beech LVL compared to poplar LVL, measurements of higher cutting forces are in accordance with expectations. All of these points show that the developed method is effective for the determination of the cutting forces.

For isotropic materials, only an average specific cutting coefficient and an average value of intercept were computed. For orthotropic materials, the cutting forces were reported every 10° of angular position/grain orientation. For beech LVL, the ratios between maximal and minimal cutting force were about two. Machining across the grain resulted in forces that were twice as large compared to machining parallel to the grain. The same ratio, even if the absolute value of the forces was lower compared to beech LVL, can be found for poplar LVL. These results are coherent with previous results found in the literature using different measurement methods. A ratio of about three was found by Kivimaa [1] for massive Birch wood; a ratio of about two was found by Goli et al. [11] for Douglas fir wood. A ratio of up to five was found by Curti et al. [13] for green beech wood and much larger chip sections.

The absolute values found for MDF and beech LVL are also coherent with the values computed using the experimental coefficients calculated by Ettelt and reported in Wagenführ and Scholz [18].

The negative values of specific cutting coefficients computed for poplar LVL could depend on the following: 1. variation in the cutting mechanics, which leads to the cutting force not increasing proportionally to the chip thickness; 2. measurement errors. The errors can be estimated to be ±5 N mm^−2^, as shown in Figure 10b when machining PTFE, which can result in much higher variations of *Ks*. According to these hypotheses, we can conclude that the *Ks* value of −12.41 N mm^−2^ observed at 100° of angular position can be explained both by variation in the cutting mechanics and errors. As shown in Figure 13a,b, a clear negative tendency of cutting forces versus depth of cut is visible for both 100° and 110° of angular position in poplar LVL. The reason for this negative value could be a different cutting mechanism.

## 5. Conclusions

The machining of a disk seems to be a very effective method to rapidly assess the specific cutting coefficients and intercepts both of isotropic and orthotropic materials. Especially for orthotropic materials, the method allows the production of oriented diagrams able to characterize a material for the whole set of possible cutting directions/grain orientations. The developed analysis has been shown to be simple and effective and the method has been shown to produce data in line with the expectations from bibliographic sources. The method can be used to produce models for the development of anisotropy adaptive machining strategies.

## Figures and Tables

**Table 1:** Machined material thicknesses and physical properties. Moisture content before machining was determined by the gravimetric method; the density of the machined samples was determined after equilibration at 20 °C and 65% relative humidity (RH).

Product	MC (%) @ Machining	Density (kg m^−3^) @20°TC65%RH	Thickness [mm]	Notes
PTFE	/	2202	21.6	
Beech LVL	9.2	724	30.1	11 plies ∥ + 2 plies ⊥ ^1^
MDF	9.3	711	30.1	
Poplar LVL	9.4	365	19.5	3 plies ∥

^1^ ∥ stands for parallel grain plies and ⊥ for perpendicular grain plies.

**Table 2:** Average specific cutting coefficient (Ks) and intercept values computed when machining PTFE with the up-milling technique.

Variable	Average (SD)	Min	Max
Ks [N mm^−2^]	20.33 (2.90)	14.46	24.81
*Int* [N mm^−1^]	2.71 (0.24)	2.40	3.15

**Table 3:** Average specific cutting coefficient (Ks) and intercept values computed when machining MDF with the up-milling technique.

Variable	Average (SD)	Min	Max
Ks [N mm^−2^]	31.44 (2.68)	25.81	35.58
*Int* [N mm^−1^]	3.36 (0.27)	2.96	3.83

**Table 4:** Specific cutting coefficient (Ks) and intercept values computed when machining beech LVL and poplar LVL with the up-milling technique at different grain orientations.

Angle	Beech LVL	Beech LVL	Poplar LVL	Poplar LVL
[°]	*Ks* [N mm^−2^]	*Int* [N mm^−1^]	*Ks* [N mm^−2^]	*Int* [N mm^−1^]
0	32.29	7.66	2.91	5.66
10	41.81	7.49	−4.78	6.18
20	44.46	7.51	9.22	5.00
30	43.00	7.23	−19.78	6.90
40	44.15	6.10	26.95	3.67
50	48.30	4.97	24.98	3.37
60	51.04	4.08	35.26	2.72
70	44.64	3.33	38.98	2.14
80	25.53	3.67	37.15	1.70
90	11.73	3.70	25.43	1.54
100	6.61	5.65	−5.62	4.05
110	14.35	6.69	−12.41	6.07
120	26.95	6.55	20.60	4.82
130	27.60	7.08	25.73	5.22
140	26.56	7.38	20.65	5.74
150	25.86	7.61	15.52	5.70
160	32.45	7.09	16.43	5.24
170	31.68	7.35	17.73	4.89
